# Thyroid, Renal, and Breast Carcinomas, Chondrosarcoma, Colon Adenomas, and Ganglioneuroma: A New Cancer Syndrome, FAP, or Just Coincidence

**DOI:** 10.1155/2016/2928084

**Published:** 2016-03-20

**Authors:** Ihab Shafek Atta, Fahd Nasser AlQahtani

**Affiliations:** ^1^Department of Pathology, Faculty of Medicine, Al-Azhar University, Assiut Branch, Assiut 71524, Egypt; ^2^Department of Radiology, Faculty of Medicine, Al-Baha University, Saudi Arabia

## Abstract

We are presenting a case associated with papillary thyroid carcinoma, renal cell carcinoma, invasive mammary carcinoma, chondrosarcoma, benign ganglioneuroma, and numerous colon adenomas. The patient had a family history of colon cancer, kidney and bladder cancers, lung cancer, thyroid cancer, leukemia, and throat and mouth cancers. She was diagnosed with colonic villous adenoma at the age of 41 followed by thyroid, renal, and breast cancers and chondrosarcoma at the ages of 48, 64, 71, and 74, respectively. Additionally, we included a table with the most common familial cancer syndromes with one or more benign or malignant tumors diagnosed in our case, namely, FAP, HNPCC, Cowden, Peutz-Jeghers, renal cancer, tuberous sclerosis, VHL, breast/other, breast/ovarian, Carney, Werner's, Bloom, Li-Fraumeni, xeroderma pigmentosum, ataxia-telangiectasia, osteochondromatosis, retinoblastoma, and MEN2A.

## 1. Introduction

A cancer diagnosed at young age, multifocal or bilateral in paired organs, more than one primary unrelated cancer, family history of cancer, or congenital anomalies will make the diagnosis of family cancers syndromes a high possibility [1]. Familial adenomatous polyposis (FAP) is a syndrome with mutations in APC gene on 5q21–q22. Diagnosis of FAP is suspected clinically in the presence of hundreds of colorectal polyps running in certain families but mutational analysis study is the golden test for diagnosis. Colon cancer, follicular cancer, papillary thyroid cancer, gastric cancer, and medulloblastomas (Turcot syndrome) are the most common malignant tumors [2, 3]. Associated benign lesions are colonic, gastric, and duodenal polyps [4, 5]. Hereditary Nonpolyposis Colon Cancer (HNPCC) is another syndrome with reported mutations in the mismatch repair gene (MMR) complexes that include several mutations as hMLH1 at 3p21.3, hMSH2 at 2p22–p21, hPMS1 at 2q31–q33, hPMS2 at 7p22, and hMSH6 at 2p16 [6]. Diagnosis depends on a family pedigree with colon cancer diagnosed before the age of 50 with no familial adenomatous polyposis detected [6]. Associated malignant neoplasms are colorectal, endometrial, ovarian, and transitional cell cancers of the urinary system, gastric cancer, small bowel cancer, pancreas cancer, hepatobiliary carcinoma, sebaceous carcinoma, glioblastoma multiforme, and breast cancer [2, 7]. Associated benign neoplasms are colonic adenomas, keratoacanthomas, sebaceous adenomas, and epitheliomas. Cowden syndrome is reported with mutations in PTEN gene at 10q23 [8–10]. Clinical diagnosis is based on mucocutaneous lesions, intestinal hamartomas, craniomegaly, coarse, dark hair, rapid overgrowth of breasts, and cerebellar glial mass leading to altered gait and seizures (Lhermitte-Duclos disease). Associated malignant tumors are breast, thyroid, colon, kidney, ovary, endometrium, and lung carcinomas, melanoma, Merkel cell skin cancer, and retinal glioma. Associated benign lesions are skin verruci of the face and limbs, hyperkeratotic papules of the gingiva and buccal mucosa, facial trichilemmomas, oral mucosal fibromas, hyperkeratosis of hand and foot, hamartomatous polyps of stomach, small bowel, and colon, lipomas, cerebellar gangliocytomatosis, thyroid adenomas, and hemangiomas [11]. Familial renal cell carcinoma (RCC) is reported with mutations in MET protooncogene at 7q31.1–34 in the papillary RCC type [12]. The gene for the nonpapillary/clear cell type located at 3p14.2 has not yet been cloned which is thought to be distinct from the gene of von Hippel-Lindau [13]. Diagnosis is mainly by finding multifocal or bilateral papillary RCC without family history or a single tumor with family history of papillary RCC [14, 15]. Associated malignant neoplasms are renal, stomach, rectal, breast, lung, pancreatic, and bile duct cancers [14, 15].

We are presenting, for the first time, a case associated with metastatic papillary thyroid carcinoma, renal cell carcinoma, invasive breast carcinoma with intraductal component, chondrosarcoma, benign ganglioneuroma, and numerous colon adenomas from the age of 41 to the age of 74.

## 2. Case Report

### 2.1. Personal History

The patient is a 76-year-old white female with past medical history significant for multiple cancers and benign tumors, hypertension, gastroesophageal reflux disease, and osteoporosis. The patient's history is significant for allergy to penicillin which causes anaphylaxis and allergy to epinephrine and oxycodone. She is nonsmoker and has one alcoholic beverage per day. She is retired and has two children. The patient is on Synthroid, Cardura, Monopril, Zocor, Prilosec, Boniva, Darvocet-N, and Colace medication.

### 2.2. Papillary Thyroid Carcinoma

**Figure 1:**
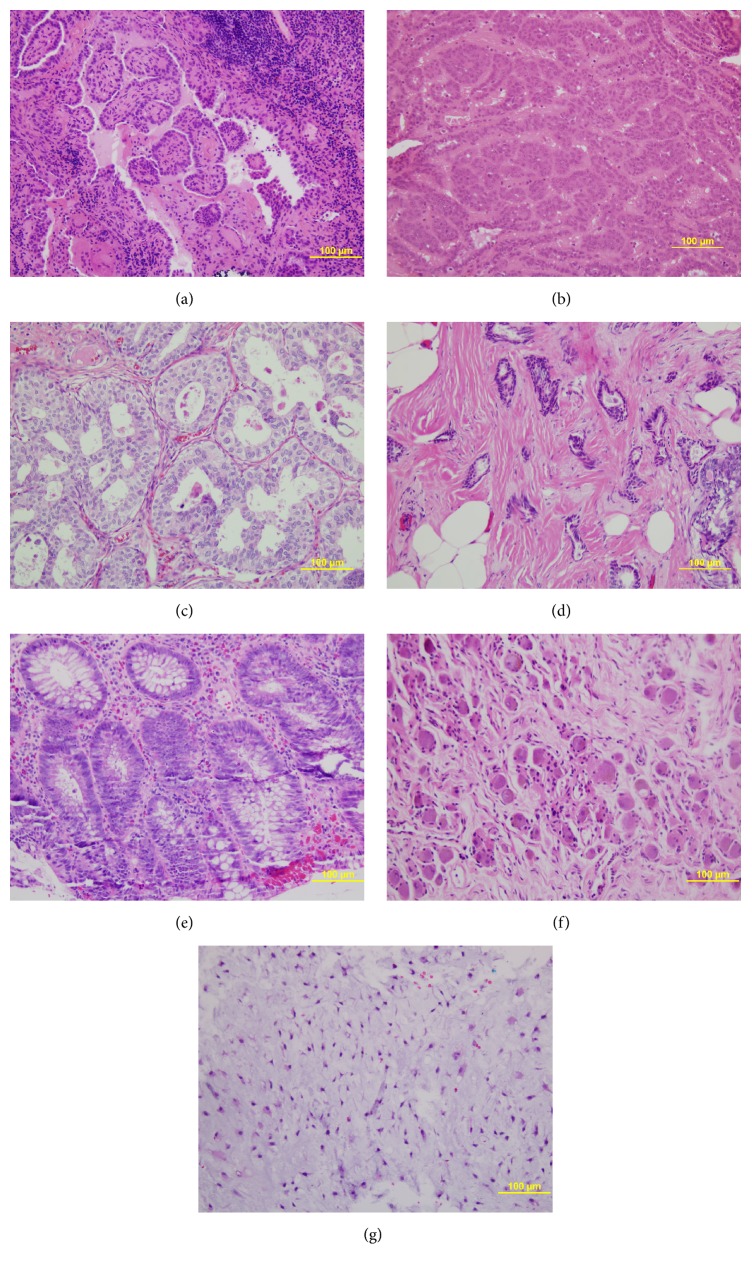
Malignant and benign lesions in the case. (a) Lymph node: metastatic papillary thyroid carcinoma in lymph node (H&E stain, ×200 original magnification, bar = 100 micrometers). (b) Kidney: renal cell carcinoma (H&E stain, ×200 original magnification, bar = 100 micrometers). (c) Breast: ductal carcinoma in situ (H&E stain, ×200 original magnification, bar = 100 micrometers). (d) Breast: invasive ductal carcinoma (H&E stain, ×200 original magnification, bar = 100 micrometers). (e) Colon: tubular adenomatous polyp (H&E stain, ×200 original magnification, bar = 100 micrometers). (f) Paratrachea: ganglioneuroma (H&E stain, ×200 original magnification, bar = 100 micrometers). (g) Chest wall: chondrosarcoma (H&E stain, ×200 original magnification, bar = 100 micrometers).

The patient had a past medical history significant for right thyroid lobectomy with modified radical ipsilateral neck dissection for papillary thyroid cancer with metastatic lymph node 28 years ago. Last year, a fine-needle aspiration and subsequent biopsy from a hypoechoic lesion in the right thyroid fossa were positive for metastatic papillary thyroid carcinoma in lymph node (Figure 1(a)) and the patient underwent reexcision followed by radioactive iodine therapy.

### 2.3. Renal Cell Carcinoma

The patient was diagnosed with moderately differentiated, 8.3 cm, Fuhrman grade 2/4 renal cell carcinoma with invasion into the perinephric fat 16 years ago requiring left nephrectomy (Figure 1(b)).

### 2.4. Mammary Carcinoma

Four years ago, the patient underwent left breast core biopsy that revealed ER/PR positive ductal carcinoma in situ (DCIS) of low-grade, cribriform pattern with microcalcification (Figure 1(c)). A ductal epithelial hyperplasia and benign papilloma were also noted. A lumpectomy specimen revealed a microscopic focus of invasive ductal carcinoma (IDC), grade I, with maximum dimension of 2 mm (Figure 1(d)) associated with multiple foci of DCIS. The IDC is ER positive and PR negative. The HER2/neu is not amplified by FISH assay. Sentinel lymph node biopsies from two lymph nodes were negative for tumor deposits.

### 2.5. Colonic Polyps and Ganglioneuroma

A large villous adenoma with focus of adenocarcinoma in situ in anterior rectal wall was excised 34 years ago, adenomatous transverse colonic adenomatous polyp and rectum hyperplastic polyp were excised 26 years ago, and a tubular adenomatous polyp in transverse colon was diagnosed 3 years ago (Figure 1(e)). Two years ago a ganglioneuroma was detected in a biopsy from paratracheal mass (Figure 1(f)).

### 2.6. Physical Examination

Physical examination and review of systems demonstrate adult female in no acute distress; she is afebrile with normal vital signs and clear oropharynx. There is no cervical adenopathy, neither carotid bruit nor jugular venous distention. Trachea is midline and lung fields are clear to auscultation bilaterally with good airflow entry.

### 2.7. Chondrosarcoma

The chondrosarcoma will be discussed here with some detail because this is the presentation of the patient prior to writing this report. The patient first noted a mass on her right chest wall last year around the time of her workup for recurrent thyroid carcinoma. Initially, the patient was asymptomatic; however, as the mass enlarged, the patient began to develop chest wall discomfort. Physical exam was significant for a palpable, fixed, nontender, 5 cm mass at the costochondral joint along the right 4th rib. The skin as well as soft tissue around the mass was freely movable. No overlying skin changes were noted. CT scan of the chest was significant for a 5.3 × 2.9 cm mass at the costochondral junction of the right 4th rib. No mediastinal lymphadenopathy or pulmonary masses were noted. A nuclear medicine bone scan revealed increased radiotracer activity along the anterior 4th rib in correlation with the findings on the CT scan. There was also increased uptake along the left 6th rib with no associated CT abnormality. Fine-needle aspiration of the mass was negative. Core needle biopsy revealed low-grade chondrosarcoma with positive ki-67 in malignant chondrocytes. The patient underwent operative excision of her chondrosarcoma with chest wall reconstruction. Postoperatively, the patient developed onset of dyspnea and hypotension and hematocrit drop from 34% to 20% and a chest X-ray revealed a right pleural fluid collection. Bleeding vessel was ligated and the hemothorax was evacuated along the breast tissue flap. Pathologically a low-grade chondrosarcoma of 6.5 cm with free soft tissue and bony margins was the diagnosis (Figure 1(g)).

### 2.8. Family History

**Figure 2:**
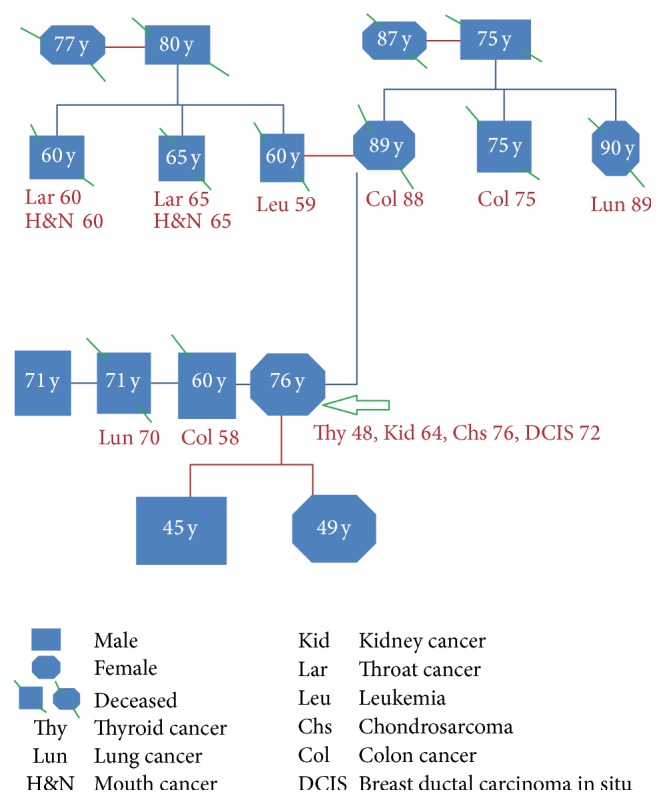
A family pedigree of the presenting case showing multiple cancers related to family members. The arrow refers to our presenting case with multiple cancers related to different body systems.

Colon cancer in sibling was diagnosed at the age of 58, patient's mother died of colon cancer at the age of 89, and two of the siblings of the patient's mother and their father were all diagnosed in their eighth or ninth decades with colon cancer. Kidney and bladder cancers were diagnosed in maternal first cousin and lung cancer was diagnosed in other brother, two maternal first cousins, and a maternal aunt. A first cousin was diagnosed with thyroid cancer. The patient's father died of leukemia at the age of 60, and each of his two brothers had throat and mouth cancers. One of the patient's brothers died of colon cancer at the age of 60, and one brother has been diagnosed with lung cancer. The patient was born in UK and there is no Jewish ancestry (family pedigree, Figure 2).

### 2.9. Molecular Genetics Investigations

Revising the chart of the patient revealed no detectable mutation of the PTEN gene by using denaturing gel gradient electrophoresis analysis. Regarding Li-Fraumeni syndrome, p53 sequencing detected no mutation in the entire coding region of the p53 gene.

## 3. Discussion

We are reporting a case with multiple cancers which could not fit in any of the reported family cancer syndromes (Table 1). The family history from the mother side is significant for colon, thyroid, lung, kidney, and bladder cancers (Figure 2) in addition to early diagnosis of large villous adenoma with focus of adenocarcinoma in situ at the age of forty-two in our case. The possibility of adenomatous polyposis coli (APC) syndrome is remote in this case because the polyposis is not constituted and no APC gene mutation analysis was performed. Additionally, the multiple cancers diagnosed in the current case look odd to what is published with FAP syndrome. The patient did not develop colon cancer mostly because of careful follow-up and surgical resections of multiple colonic adenomas.

The most common associated cancers published so far with FAP syndrome are colon, ampullary, and duodenal cancers, follicular or papillary thyroid carcinoma, hepatoblastomas in children, gastric cancer, and NS medulloblastomas. Associated benign lesions are colonic, gastric, and duodenal polyps [2, 4, 5, 7], epidermoid and sebaceous cysts, lipomas, osteomas, and desmoid tumors [16, 17]. In addition to what is in the literature this case had renal cell carcinoma, breast carcinoma, chest wall chondrosarcoma, and ganglioneuroma.

To exclude the possibility of Cowden syndrome associated with breast, thyroid, especially follicular type, colon, and kidney lesions besides many other malignant and benign lesions (Table 1), no mutations were detected in the PTEN gene at 10q23 in our case [8–10]. Additionally, the p53 gene is not mutated to exclude to great extent Li-Fraumeni syndrome which is associated with breast cancer and any other invasive cancer early in life compared to general population with known mutations in the p53 gene at 17p13.1 [18–21].

Table 1 summarizes mode of inheritance and tumors associated with family cancer syndromes with one or more components from the tumor constellation of our case: papillary thyroid carcinoma, renal cell carcinoma, breast carcinoma, chondrosarcoma, colonic adenomas, and ganglioneuroma. So, are we facing a case with constellation of one of the family cancer syndromes listed in the table, just a coincidence, or a new cancer syndrome?

## Figures and Tables

**Table 1:** Most common reported cancer syndromes with one or more components from the presented case.

Syndrome	Inheritance	Thyroid carcinoma	Renal carcinoma	Breast carcinoma	Chondrosarcoma	Colonic adenomas	Ganglioneuroma
Adenomatous polyposis	Dominant	X				X	
Colon (HNPCC)	Dominant		X	X		X	
Cowden	Dominant	X	X	X			
Peutz-Jeghers	Dominant			X		X	
Renal cancer	Dominant		X	X			
Tuberous sclerosis	Dominant	X	X				
VHL	Dominant		X				
Breast/other	Dominant	X	X	X	X		
Breast/ovarian	Dominant			X			
Carney	Dominant	X					
Werner's	Recessive	X		X			
Bloom	Recessive			X			
Li-Fraumeni	Dominant			X			
Xeroderma pigmentosum	Dominant			X			
Ataxia-telangiectasia	Recessive			X			
Osteochondromatosis	Dominant				X		
Retinoblastoma	Dominant				X		
MEN2A	Dominant						X
